# A Holistic Well-being Approach for Healthcare Staff in Long-term Ventilation Care Settings

**DOI:** 10.1093/geroni/igaf122.1155

**Published:** 2025-12-31

**Authors:** Sharon Ost-Mor, Dikla Segel-Karpas, Jonathan Sarig, Gizelle Green, Tammi Packer-Porat

**Affiliations:** University of Haifa, Haifa, HaZafon, Israel; University of Haifa, Haifa, HaZafon, Israel; State University of Florida, Jacksonville, Florida, United States; Shoham Medical Center, Pardes-Hanna, HaMerkaz, Israel; Shoham Medical Center, Pardes-Hanna, HaMerkaz, Israel

## Abstract

“Positive Solitude (PS) is a novel concept that redefines alone time as significant, meaningful, and potentially beneficial for healthcare workers facing stress in ventilation care units. This study examines healthcare professionals in long-term ventilation care settings, exploring the potential benefits of PS interventions in mitigating stress and promoting well-being in both professional and personal contexts. Using a qualitative descriptive research approach, a six-week PS intervention was implemented among 24 multidisciplinary staff members in the ventilation ward, incorporating structured alone time, mindfulness, and reflectiveness. Two groups of 12 participants were recruited from two similar ventilation wards. The intervention aimed to foster self-awareness, resilience, and coping strategies among participants by encouraging intentional and meaningful alone time, with qualitative interviews conducted pre- and post-intervention. Analysis of the interviews revealed themes including improved work-life balance, heightened awareness of PS benefits, and enhanced stress management skills. The intervention group reported significant improvements in using PS skills compared to the control group, though the control group reported increased awareness of their PS in general. This study contributes theoretically to understanding the advantages of PS and provides practical insights for supporting healthcare professionals’ well-being and occupational experiences in high-stress environments. The findings highlight the potential of PS interventions in promoting resilience among healthcare staff, with implications for improving both worker well-being and quality of care.

